# Identification of candidate genes related to pancreatic cancer based on analysis of gene co-expression and protein-protein interaction network

**DOI:** 10.18632/oncotarget.20537

**Published:** 2017-08-24

**Authors:** Tiejun Zhang, Xiaojuan Wang, Zhenyu Yue

**Affiliations:** ^1^ GMU-GIBH Joint School of Life Sciences, Guangzhou Medical University, Guangzhou, Guangdong 511436, China; ^2^ Institute of Health Sciences, School of Computer Science and Technology, Anhui University, Hefei, Anhui 230601, China

**Keywords:** pancreatic cancer, candidate genes, gene co-expression, protein-protein interaction network, subnetwork extraction algorithm

## Abstract

Pancreatic cancer (PC) is one of the most common causes of cancer mortality worldwide. As the genetic mechanism of this complex disease is not uncovered clearly, identification of related genes of PC is of great significance that could provide new insights into gene function as well as potential therapy targets. In this study, we performed an integrated network method to discover PC candidate genes based on known PC related genes. Utilizing the subnetwork extraction algorithm with gene co-expression profiles and protein-protein interaction data, we obtained the integrated network comprising of the known PC related genes (denoted as seed genes) and the putative genes (denoted as linker genes). We then prioritized the linker genes based on their network information and inferred six key genes (*KRT19*, *BARD1*, *MST1R*, *S100A14*, *LGALS1* and *RNF168*) as candidate genes of PC. Further analysis indicated that all of these genes have been reported as pancreatic cancer associated genes. Finally, we developed an expression signature using these six key genes which significantly stratified PC patients according to overall survival (Logrank *p* = 0.003) and was validated on an independent clinical cohort (Logrank *p* = 0.03). Overall, the identified six genes might offer helpful prognostic stratification information and be suitable to transfer to clinical use in PC patients.

## INTRODUCTION

Pancreatic cancer (PC) is a common type of cancer with an estimated 53,070 new cases and 41,780 deaths expected in the United States in 2016 [1]. In contrast to most cancers, the 5-year relative survival of PC patients which is currently 8% increases slowly [1]. Risk factors for developing PC include tobacco smoking, obesity, diabetes, and certain rare genetic conditions. Several mutations in *PIK3CA*, *PALB2* and *TP53* involve in the carcinogenesis of PC. However, these mutations account for only a fraction of the total PC burden.

The types of PC can be divided into two general groups based on histological origin, i.e. the majority and minority group occurring in exocrine and endocrine component of pancreas, respectively. Usually by the time of diagnosis, pancreatic cancer has often spread through the body due to less symptoms in the disease's early stages. Generally, 25% of people survive one year and 5% live for five years after diagnosis [2]. Genomic analyses of PC reveal a complex molecular landscape. And numerous events have been found involved in pancreatic tumorigenesis which can be acted as signatures for diagnosis and further clinical application, such as mutations found in well-known cancer genes (*KRAS*, *TP53*, *SMAD4*, and *CDKN2A*) [3]. Among these, Mutations in *SMAD4* are especially associated with a worse prognosis [4]. Despite significant improvements in the early molecular diagnosis and treatment decisions of PC, new therapies and biomarkers are still needed.

At present, although a variety of relevant genes have been identified, they are insufficient to elucidate the tumorigenesis of PC unless more related genes being uncovered. Therefore, it is an extremely critical task to discover novel candidate genes. It is time-consuming and cost-spending to find disease related genes by experiment alone on account of the vast search space. Computational approach is an alternative solution which can assist researchers to cope with a mount of biological problems, for instance, to clarify complicated biological network [5–9] and to identifycandidate genes [10, 11]. For example, Huang et al. presented a model for inferring protein-protein interactions based on protein-protein correlation utilizing least squares regression [9]. Zhu et al. proposed a robust geometric approach for modeling protein-protein interaction networks [8]. Deng et al. developed a method to predict novel genes associated with cervical carcinoma using gene co-expression networks [10]. Moreover, several studies have reported prognostic genomic signatures for PC [12–14]. Stratford et al. analyzed the gene expression profiles of primary PC patients from localized tumors compared to metastatic ones and identified a six-gene signature associated with metastatic PC [12]. Haider et al. performed a retrospective meta-analysis on publicly available mRNA abundance datasets to discover gene signatures based on differentially expressed genes and univariate prognostic gene selection [13]. Chen et al. derived a prognostic gene signature for patients with early stage pancreatic ductal adenocarcinoma using sparse principal component analysis and univariate analysis of Cox proportional hazards model [14]. However, these studies have generally been limited by only gene expression analysis and particular PC subtypes. Thus, presentation of a more robust molecular predictor that incorporates multi-omics data and overcomes PC subtype variability will undoubtedly have an important role in the management of patients as well as identification of novel genes.

In this research, a computational approach was proposed to discover PC candidate genes based on known PC related genes retrieved from PCGene (Pancreatic Cancer Gene Database, http://pcgene.bioinfo-minzhao.org) [15]. Utilizing the subnetwork extraction algorithm with gene co-expression profiles and protein-protein interaction (PPI) data, we obtained the network comprising of the known PC related genes (denoted as seed genes) and the putative genes (denoted as linker genes). After prioritizing the linker genes on the basis of subnetwork information, we found top-six valuable candidate genes. Further analysis indicates that all of these six genes are in accord with previous reports that they are implicated with PC or cancers. Using the 6-genes signature, we observed significant differences in overall survival between low-risk and high-risk PC patients in survival analyses of both training and independent test datasets. Overall, our analysis developed significant insight that will lead to improved prognostication and risk stratification of PC patients.

## RESULTS AND DISCUSSION

### Strategy for prediction of pancreatic cancer candidate genes

**Figure 1:**
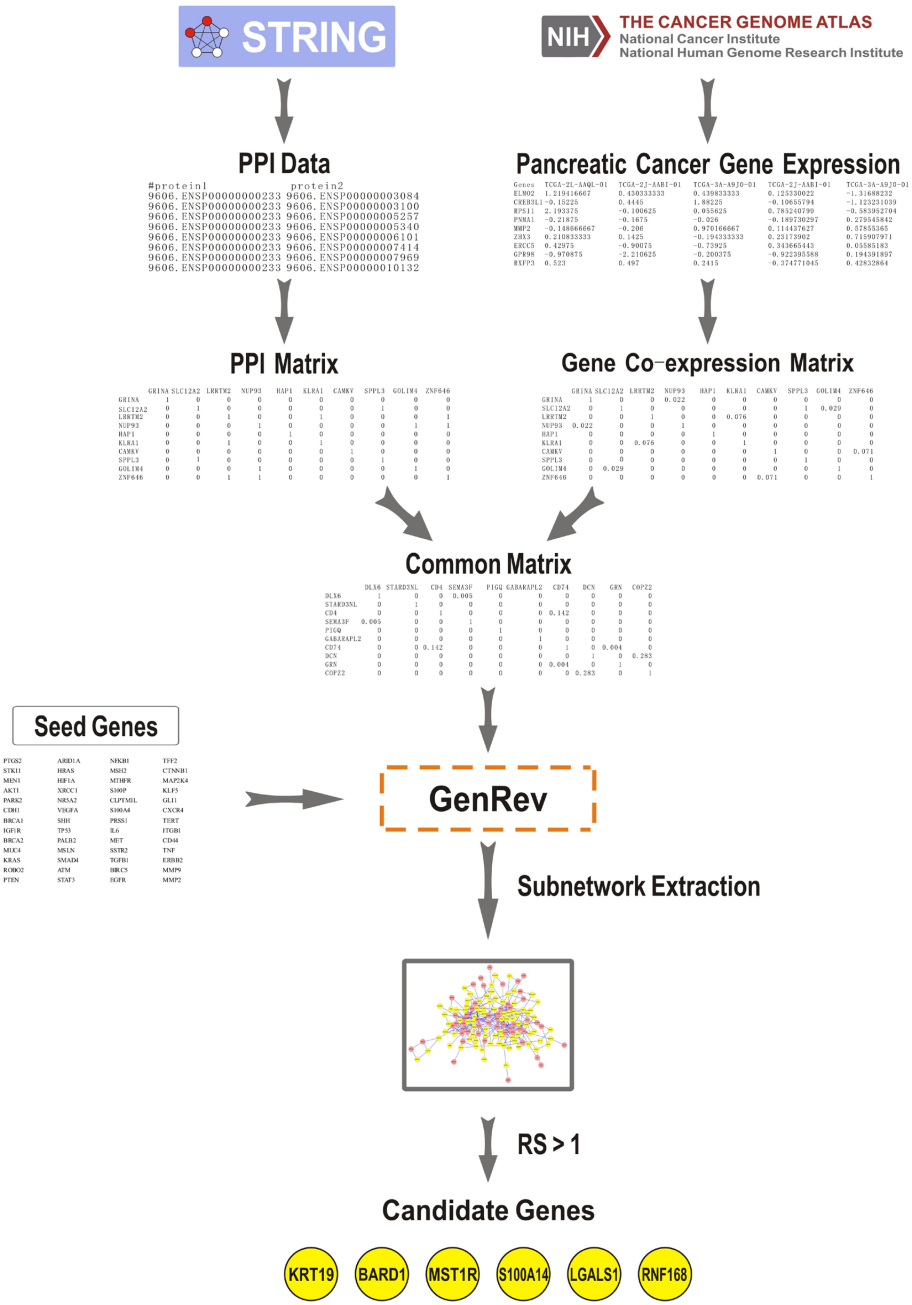
Summary workflow for prediction of pancreatic cancer candidate genes The approach was based on three steps: (1) We constructed a PPI network and a co-expression network using data derived from STRING (Search Tool for the Retrieval of Interacting Genes/Proteins) and The Cancer Genome Atlas (TCGA), respectively. A common network was obtained through comparing the two networks. (2) Seed genes and the common network were imported into GenRev which was used to extract a subnetwork with the limited k-walks algorithm. (3) 6 key candidate genes were identified among the subnetwork using an evaluation indicator denoted as ranking score (RS).

This work was aimed at using seed genes and a common biological network containing gene co-expression and PPI information to discover candidate genes related to the pathogenesis of PC with a subnetwork extraction algorithm. The method mainly divided into three steps as follows (Figure 1).

(1) A human PPI network was obtained from STRING (Search Tool for the Retrieval of Interacting Genes/Proteins), which is a database of verified and predicted protein interactions [16]. And then we collected gene expression data of 179 PC tumor samples derived from The Cancer Genome Atlas (TCGA) using UCSC Cancer Genomics Browser [17]. The co-expression network was constructed by an R software package named “WGCNA” [18]. Through comparing the PPI network with the gene co-expression network, a common gene network containing both information types was generated. Table 1 illustrated the number of nodes and edges in PPI network, co-expression network and common network.

**Table 1:** Numbers of nodes and edges in networks

	PPI Network	Co-expression Network	Common Network
Nodes	17865	16226	12716
Edges	8548003	451034	225517

(2) 52 seed genes were collected from PCGene, after removing 11 genes which are not included in the common network (Table 2). Seed genes and the common network were then inputted into GenRev software [19]. We used the limited k-walks algorithm in GenRev for extracting a subnetwork (see more details in the Materials and Methods section).

**Table 2:** Seed genes used in this present study

Gene names			
PTGS2	ARID1A	NFKB1	TFF2
STK11	HRAS	MSH2	CTNNB1
MEN1	HIF1A	MTHFR	MAP2K4
AKT1	XRCC1	S100P	KLF5
PARK2	NR5A2	CLPTM1L	GLI1
CDH1	VEGFA	S100A4	CXCR4
BRCA1	SHH	PRSS1	TERT
IGF1R	TP53	IL6	ITGB1
BRCA2	PALB2	MET	CD44
MUC4	MSLN	SSTR2	TNF
KRAS	SMAD4	TGFB1	ERBB2
ROBO2	ATM	BIRC5	MMP9
PTEN	STAT3	EGFR	MMP2

(3) In this step, we identified 6 key candidate genes among the 82 linker genes derived from the subnetwork using an evaluation indicator denoted as ranking score (RS), which is the sum of weights of all edges linked to a particular gene. In order to underline the linkage between a seed gene and a linker gene, the weight of them is doubled when calculated. RSs of all the 82 linker genes were listed in Table 3. Then we analyzed these 6 genes (RS > 1) by retrieving existing literature and estimated the prognostic value of this 6-genes signature using the transcriptomic data by survival analysis.

**Table 3:** The 82 linker genes with their ranking scores

Gene names	RS	Gene names	RS	Gene names	RS
KRT19	1.6	PTGES2	0.4	BCL7C	0.2
BARD1	1.6	PREX2	0.4	ZNF18	0.2
MST1R	1.5	USP1	0.4	PGLS	0.2
S100A14	1.2	EHF	0.4	CNTN4	0.2
LGALS1	1.2	ADM	0.4	BACH1	0.2
RNF168	1.1	GRM4	0.4	ZFPL1	0.2
TFF1	1	SHFM1	0.4	MAGED2	0.1
SPEN	1	EMP3	0.4	TMEM43	0.1
WDR18	0.9	PTGES	0.3	GALNT5	0.1
RRAS	0.9	TRAF1	0.3	HMX1	0.1
PLEK2	0.8	NAMPT	0.3	RASSF6	0.1
CD248	0.8	BCL2L12	0.3	PFN1	0.1
RHBDL2	0.8	MAML1	0.3	SLC16A7	0.1
CAPN5	0.7	USP24	0.3	ZBTB40	0.1
S100A16	0.7	AMHR2	0.3	MAGED4	0.1
CDC6	0.6	PSMG3	0.3	PIF1	0.1
ENDOG	0.6	NFE2L2	0.3	MBTPS1	0.1
GNG2	0.6	RAPH1	0.3	HDAC4	0.1
CLDN18	0.6	TSHZ2	0.2	VASN	0.1
RNF169	0.5	RNF146	0.2	PSME3	0.1
NR3C1	0.5	NFIL3	0.2	AMN	0.1
TMC8	0.5	SPINK1	0.2	ERCC1	0.1
AKT1S1	0.5	OSTM1	0.2	BRD9	0.1
KLK10	0.5	ESRRG	0.2	B4GALNT1	0.1
SLC2A1	0.5	XRCC3	0.2	PLEKHG5	0.1
ANTXR1	0.4	SLC4A7	0.2	ARHGAP33	0.1
CHSY1	0.4	DPH1	0.2	CYP24A1	0.1
ELAC1	0.4				

RS, ranking score.

Figure 1 illustrated a summary workflow for identifying genes critical to PC.

### Subnetwork and pancreatic cancer candidate genes

As described in the Methods section, proteins or genes in a same biological (PPI or co-expression) network may share some common or similar features. Thereby, after importing seed genes and the common network that represented both gene co-expression and PPI information into GenRev software, we extracted a subnetwork connecting high-confident PC related genes by edge-weighted limited k-walk algorithm [20].

**Figure 2:**
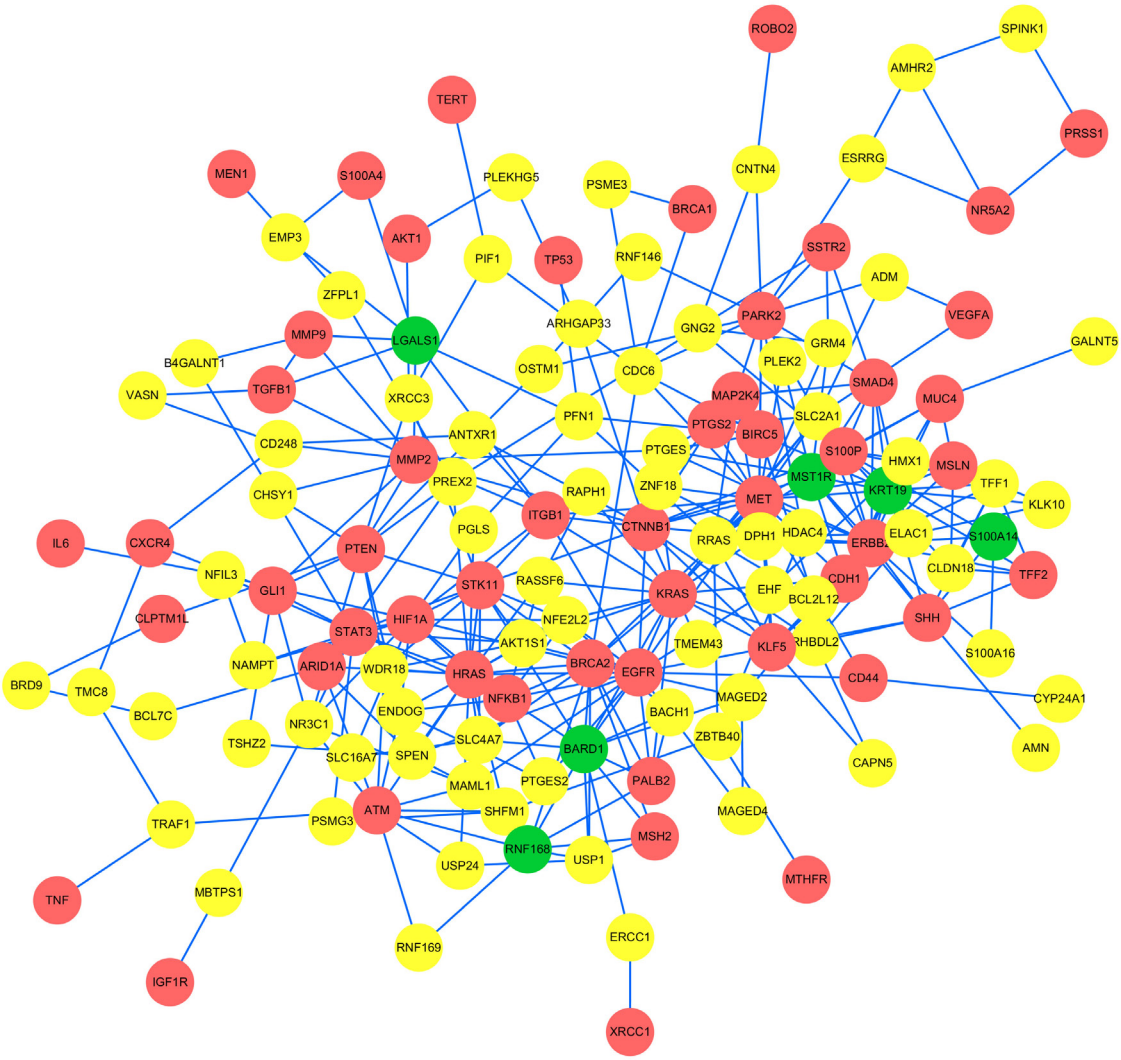
The subnetwork extracted by limited k-walks algorithm The network extracted by edge-weighted limited k-walk algorithm was illustrated. It contained 341 edges and 134 nodes. Red nodes represent 52 seed genes, green nodes represent 6 identified candidate genes and yellow nodes represent the other 76 linker genes except for the 6 candidates.

The network extracted by edge-weighted limited k-walk algorithm was illuminated in Figure 2 which contained 341 edges and 134 nodes. Except for 52 seed genes, there are 82 linker genes included in the subnetwork. Then we obtained 6 key candidate genes from the 82 linker genes using the ranking score defined, which is a prioritization strategy of candidate genes.

### Literature analysis of pancreatic cancer candidate genes

**Figure 3:**
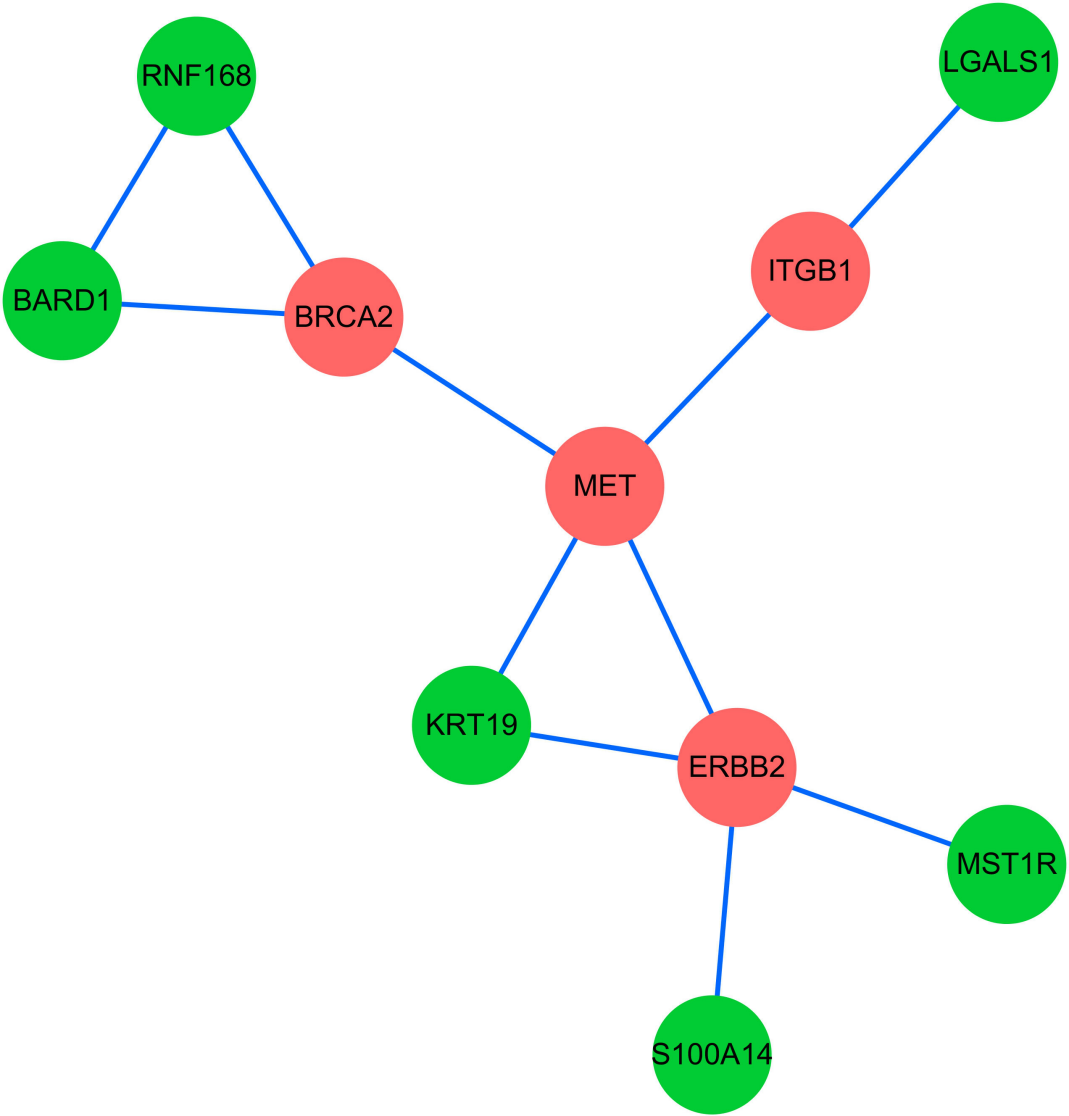
The smallest subnetwork connecting six key candidate genes derived from the extracted network Red nodes represent 4 seed genes and green nodes represent 6 identified candidate genes.

After ranking the linker genes from the extracted subnetwork, we obtained 6 genes which might have a strong relationship with PC. We identified the smallest subnetwork connecting six key genes derived from the network representing both PPI and gene co-expression information (Figure 3). The other four genes in this subnetwork are all PC related seed genes (*BRCA2*, *MET*, *ITGB1*, *ERBB2*). Succeeding analysis manifested that all of these six key genes (*KRT19*, *BARD1*, *MST1R*, *S100A14*, *LGALS1* and *RNF168*) have been shown as PC associated genes based on previous reports. Next, these 6 genes will be elaborated one after another in the following paragraphs.

The *KRT19* is the gene encodes a member of the keratin family which is intermediate filament protein responsible for the structural integrity of epithelial cells and consists of cytokeratins and hair keratins. It is expressed in the periderm, the transiently superficial layer that envelopes the developing epidermis. Previous study has revealed that it can be a marker gene as a way to detect circulating tumor cells in peripheral blood of pancreatic cancer patients [21].

The protein encoded by *BARD1* interacts with the N-terminal region of *BRCA1* which is a tumor suppressor gene found in various cancers [22, 23] and shares homology with the conserved regions of it (the N-terminal RING motif and the C-terminal BRCT domain). An allele-specific expression assay have supported that this is a candidate PC related gene with altered germline expression properties as compared to controls [24]. In addition, Brakeleer et al. provided evidence for an increased breast cancer risk associated to specific *BARD1* germline mutations [25].

The *MST1R* gene encodes a cell surface receptor for macrophage-stimulating protein (MSP) with tyrosine kinase activity which has been identified as an important mediator of *KRAS* oncogene addiction and is over-expressed in the majority of pancreatic cancers. Inhibition of its function decrease pancreatic cancer cell migration, invasion and survival and can sensitize PC cells to chemotherapy on the basis of preclinical studies [26, 27].

The protein encoded by *S100A14* is a member of the S100 protein family which contains an EF-hand motif and binds calcium. Proteins in this family, including S100A14, have a broad range of intracellular and extracellular functions that regulate multiple cellular pathways related to pancreatic cancer progression and metastasis [28, 29]. Such as, Intracellular S100A14 may promote cell motility and invasiveness by regulating the expression and function of matrix metalloproteinase-2 (MMP-2) in a p53-dependent manner.

The *LGALS1* encodes the galectins which belong to a family of beta-galactoside-binding proteins involved in modulating cell-cell and cell-matrix interactions. It has been reported that the expression of this gene might provide insights into prognostication for resectable pancreatic ductal adenocarcinoma. Analyses of very long-term survivors (VLTS) compared with short-term ones confirmed that significantly lower expression of stromal galectin-1 was associated with VLTS [30, 31].

The protein encoded by *RNF168* is an E3 ubiquitin ligase protein that contains a RING finger, a motif present in functionally distinct proteins and known to be implicated in DNA double-strand break (DSB) repair and protein-protein interactions. In a recently published study, Slavin et al. evaluated a set of genes for their mutation profile in pancreatic adenocarcinomas. 27 genes had truncating variants identified in patients from a hereditary pancreatic cancer cohort, including a stop-gain variant at position 131 (Arg) in RNF168, which may be related to hereditary PC predisposition [32]. Kongsema et al. demonstrated that the expression level of *FOXM1* reduced upon *RNF168* overexpression and increased with *RNF168* depletion by siRNA. And further experiments suggested that this enzyme cooperates with RNF8 to mediate FOXM1 ubiquitination and degradation in breast cancer epirubicin treatment [33]. Together, these six key genes are shown to play a direct or indirect role in PC according to the existing literature and deserve further investigation concerned with their application against PC.

### Correlation analysis of pancreatic cancer candidate genes

To analysis correlations between the expression of identified candidate genes and their corresponding seed genes, we computed the Pearson correlation coefficients using the expressions in 179 cancer samples from TCGA. As expected, all the 34 seed-candidate pairs in the extracted subnetwork have significant correlations with each other since the co-expression information has been incorporated. The coefficients and P-values are listed in Supplementary Table 1.

We also performed the correlation analysis of the 6 candidate genes with some clinical features of the PC patients. It turned out that gene expressions of the 6 candidates are not influenced by gender, alcohol and smoking history. For instance, as illustrated in Supplementary Table 2, there is no significant difference between the male and female group for each of genes according to t-test. The same scenario is presented considering either alcohol or smoking history.

### Survival analysis of pancreatic cancer candidate genes

**Figure 4:**
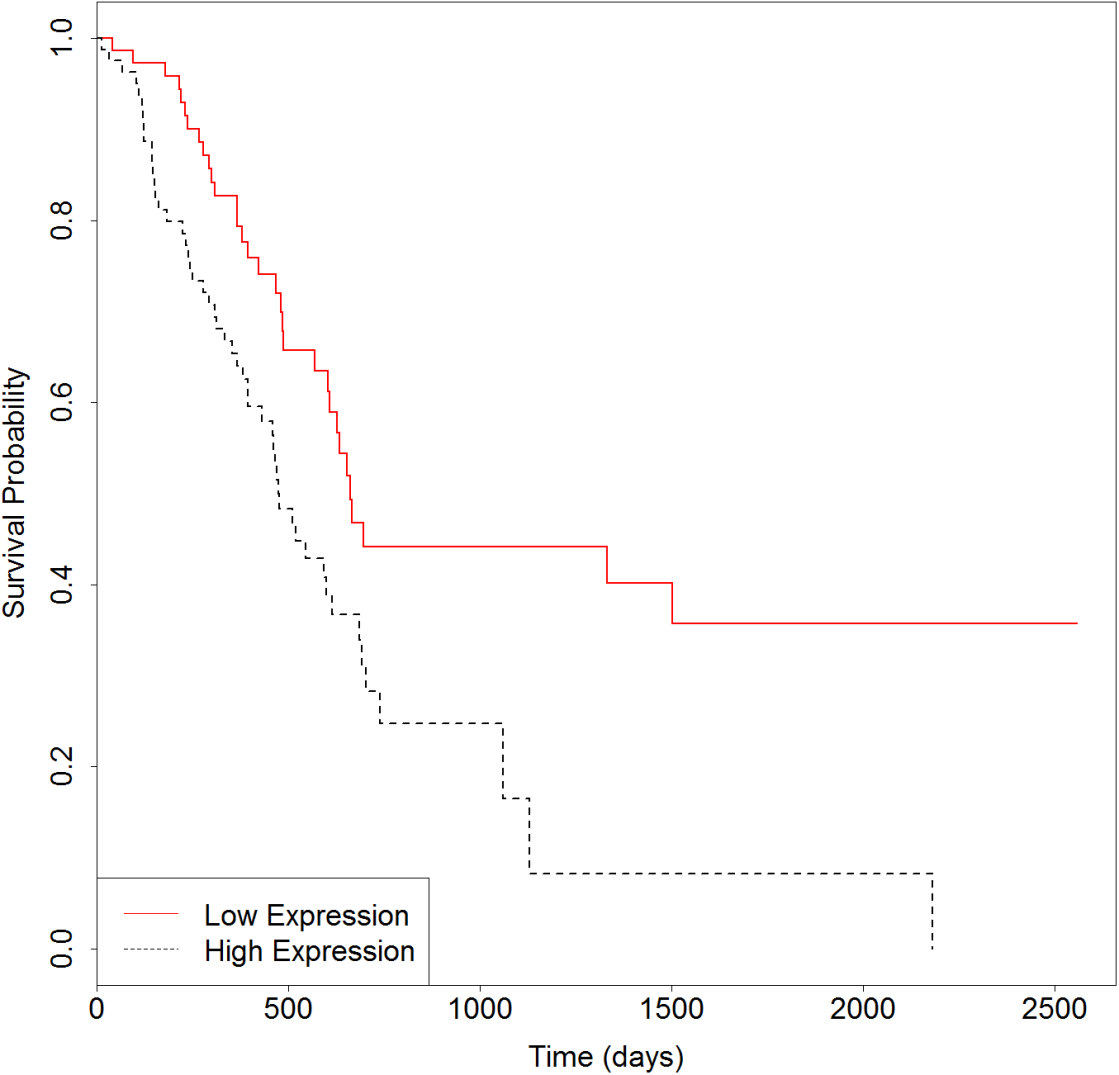
Survival plot of overall survival in samples from TCGA using the 6-genes signature Black dotted line depicts genes with higher expression associated with poor overall survival and red solid line depicts genes with lower expression associated with good survival.

**Figure 5:**
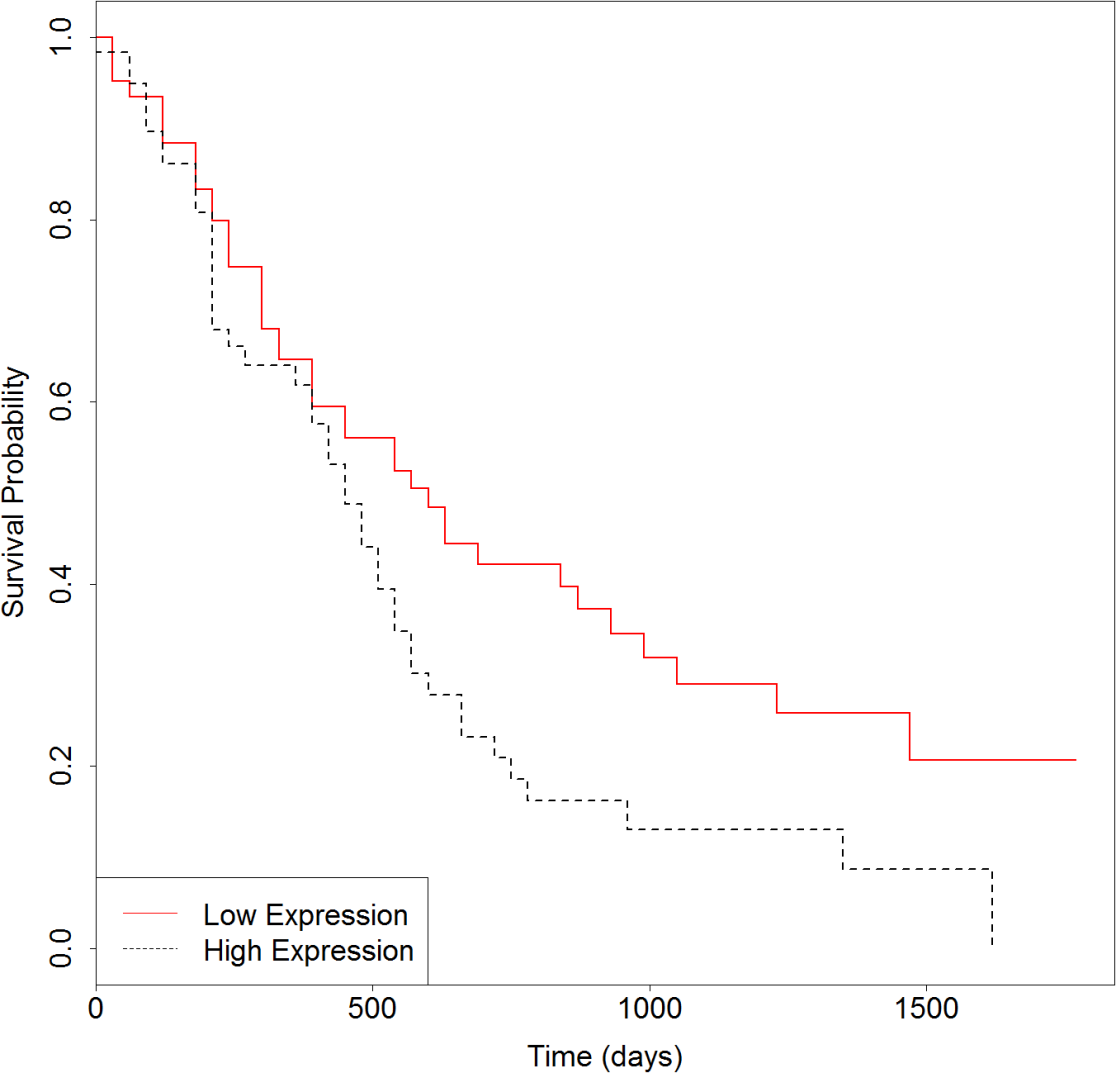
Survival plot of overall survival in samples from Moffitt et al. using the 6-genes signature Black dotted line depicts genes with higher expression associated with poor overall survival and red solid line depicts genes with lower expression associated with good survival.

We then developed an expression signature consisting of the six genes by survival analysis in R software. After removing 16 patients whose clinical data are not included in TCGA, this 6-genes signature could significantly stratify 163 PC patients according to overall survival (Logrank *p* = 0.003). As seen in Figure 4, the low-risk group had significantly better overall survival than the high-risk group. In consistent with the known function of these genes, particularly of *MST1R*, *S100A14* and *LGALS1*, the low-risk group is exactly the half with lower expressions. For further validation of the 6-genes signature, we obtained gene expression and clinical data of 125 primary pancreatic cancers from Moffitt et al. [34]. When applied to this independent test set, the result in Figure 5 (Logrank *p* = 0.03) suggested that the biomarker is able to prognosticate PC independent of patient cohorts and sequencing platforms. Further work on the functional actions and downstream events of these genes is likely to uncover true genomic candidates for PC therapeutics.

In conclusion, the identified six candidate genes from this study provide a better understand for the underlying molecular mechanisms of PC and might be useful for possible personalized therapeutic regimen selection to improve survival. It has not escaped our notice that this approach may facilitate a further exploration of PC as well as other cancers. In addition to the PPI and gene co-expression information concerned in this study, integrated genomic analysis, such as incorporation of epigenetic and mutation level information, might play a greater role in diagnosis, prognosis and therapy of PC. Furthermore, we will identify driver mutations that confer a selective growth advantage of PC cells using these multidimensional data sets in the future work.

## MATERIALS AND METHODS

### Protein-protein interaction network

Protein-protein interaction (PPI) networks carry a large amount of valuable information to understand cellular function and biological events. Numerous researches have indicated that the two adjacent proteins of the same interaction in the PPI network usually have some common features [35–38]. It can be further deduced that proteins in the subnetwork linking known PC related genes have more possibility to share similar biological functions as demonstrated in many studies [39, 40]. In this study, the PPI network was developed based on the protein-protein interaction derived from a well-known online interaction repository, STRING (Search Tool for the Retrieval of Interacting Genes/Proteins, http://string-db.org/) (9606.protein.links.v10.0) [16], which includes direct physical and indirect functional relations.

### Gene co-expression network

For the past few years, gene co-expression network has presented as a new scheme for transcriptome analysis [41, 42]. The expression levels of two co-expressed genes always go up and down synchronously. It has been shown that functionally associated genes are frequently co-expressed forming conservative transcription modules [43, 44]. Here, gene expression data of 179 PC tumor samples from The Cancer Genome Atlas (TCGA, http://cancergenome.nih.gov) were downloaded using UCSC Cancer Genomics Browser. TCGA is a project aimed to generate comprehensive, multi-dimensional maps of the key genomic changes in major types and subtypes of cancer [45]. UCSC Cancer Genomics Browser is an online interactive genome browser hosted by the University of California, Santa Cruz, which offers access to genome sequence data (https://genome-cancer.ucsc.edu/proj/site/hgHeatmap/) [17]. Then a gene co-expression network was developed in R software. Through package “WGCNA” (Weighted correlation network analysis) which is a comprehensive collection of R functions, we performed the weighted correlation network analysis [18]. The similarity scores were evaluated to denote the distance between each pair of genes using the function “adjacency ()”. Pearson's correlation coefficient is acted as the co-expression measure. The number of nodes and edges in PPI network, co-expression network and common network, respectively, were illustrated in Table 1.

### Seed genes

PC related genes were collected from the PCGene Database (http://pcgene.bioinfo-minzhao.org) which is a literature-based knowledgebase of pancreatic cancer related gene [15]. After removing 11 gene which are absent in the common network representing both PPI and gene co-expression information, we obtained 52 genes as PC related genes (seed genes) with a high level of confidence (Table 2).

### Subnetwork extraction

In this study, we aimed to propose a protocol to discover candidate genes related to PC through constructing a common network contained both protein interaction and gene co-expression information. To date, there are a number of methods that can be used to find the subnetworks. Here, a standalone and platform independent software named GenRev [19], which is able to explore the functional relevance genes, was used to extract subnetwork. The input documents contained seed genes and common network. The Pearson's correlation coefficients in co-expression network were used as edge weights. Because the common network imported into GenRev has only weights of edges but not weights of all nodes, we used the suitable edge-weighted limited k-walk algorithm in GenRev. As results, by GenRev the genes were mapped to the common network and the subnetwork was extracted. Visualization of the subnetwork and the linker genes were implemented using Cytoscape software [46].

### Limited k-walks algorithm

The limited k-walks algorithm is one of three subnetwork extraction algorithms in GenRev, which can run randomly in the network by utilizing a Markov chain and construct a relevant subnetwork connecting seed nodes. The relevance of an edge and a node associated with the seed genes is calculated through the expected times random walk passes starting from one seed node to any of the others. Here, the Pearson's correlation coefficients in co-expression network were set as weights of edges when the limited k-walk algorithm was used. More details with respect to it are available in the original work [20].

### Prioritization of candidate genes

The network extracted by edge-weighted limited k-walk algorithm contained 134 genes. Among them, 82 linker genes are considered as the putative genes. For each of the 82 linker genes, we calculated the sum of all edge weights in the subnetwork denoted as ranking score (RS). Due to linkage between a seed gene and a linker gene is of greater meaning than that between two linker genes, we doubled the edge weights of linker and seed genes when calculated the RSs. A larger ranking score of a gene signifies that it might be more likely to significant in PC. In consequence, the top-six genes which are deemed to key candidate genes of PC were obtained when the RS cutoff greater than 1 was used. The 82 linker genes with their ranking scores were listed in Table 3.

### Survival analysis of candidate genes

In this study, an R package “survival” (Survival Analysis) which contains the core survival analysis routines, including a series of survival models [47], was used to assess prognosis value of the signature developed by the six key candidate genes. To analyze the prognostic ability of 6 genes, excluding 16 patients whose clinical data are absent in TCGA, the remaining 163 overall survival samples were split into two groups according to the median of sums of the proposed 6 gene expressions. The two patient cohorts were compared by survival curves using the function “survfit ()”. Meanwhile, *p-*value of Log-rank test was calculated using the function “coxph ()”. The same method of dichotomy was applied to the 125 overall survival samples in the independent test dataset [34].

## SUPPLEMENTARY MATERIALS TABLES





## Abbreviations

PC: Pancreatic cancer
PCGene: Pancreatic Cancer Gene
PPI: Protein-protein interaction
TCGA: The Cancer Genome Atlas
RS: Ranking score
