# Supplementary material for: Identification of candidate genes related to pancreatic cancer based on analysis of gene co-expression and protein-protein interaction network
Source: Oncotarget. 2017 Aug 24;8(41):71105–16. doi: 10.18632/oncotarget.20537 (PMC5642621; doi:10.18632/oncotarget.20537)
Supplement: Supplementary file 1 [file oncotarget-08-71105-s001.pdf]

Identification of candidate genes related to pancreatic cancer based on analysis of gene co-expression and protein-protein interaction network

SUPPLEMENTARY MATERIALS

Supplementary Table 1: Pearson correlation coefficients between the expression of the 6 candidate genes and their corresponding seed genes

See Supplementary File 1

Supplementary Table 2: Correlation analysis of the 6 candidate genes with gender, alcohol and smoking history

Clinical features	BARD1	S100A14	MST1R	KRT19	RNF168	LGALS1
Male	-0.1936706	2.910202	3.526002	3.965755	-0.070963473	0.5955816
Female	-0.1930404	2.65148	3.372016	3.861836	0.002650813	0.8130857
P-value	0.9973	0.4898	0.6021	0.7231	0.5468	0.1974
alcohol(Yes)	-0.2309503	2.796914	3.483311	4.012401	-0.08918733	0.7136621
alcohol(No)	-0.1576431	2.783906	3.445058	3.79862	-0.01558954	0.6229395
P-value	0.7255	0.9745	0.9038	0.5122	0.5881	0.6113
smoker	-0.1477071	2.85957	3.663028	4.100958	-0.00385852	0.732595
non-smoker	-0.4100906	2.606145	3.1	3.778681	-0.18000173	0.7088596
P-value	0.224	0.5859	0.1276	0.3756	0.2165	0.9073

The P-value is computated using t-test, whereas the other values in the table are means of gene expression for each clinical feature group.
